# Multifunctional Properties of Quercitrin-Coated Porous Ti-6Al-4V Implants for Orthopaedic Applications Assessed In Vitro

**DOI:** 10.3390/jcm9030855

**Published:** 2020-03-20

**Authors:** Maria Antonia Llopis-Grimalt, Aina Arbós, Maria Gil-Mir, Aleksandra Mosur, Prathamesh Kulkarni, Armando Salito, Joana M. Ramis, Marta Monjo

**Affiliations:** 1 Group of Cell Therapy and Tissue Engineering, Department of Fundamental Biology and Health Sciences, Research Institute on Health Sciences (IUNICS), University of the Balearic Islands, 07122 Palma, Spain; mantonia.llopis@uib.es; 2 Health Research Institute of the Balearic Islands (IdISBa), 07010 Palma, Spain; 3 NuMat Medtech, S.L., 07121 Palma, Spainmaria.gil@numat.es (M.G.-M.); 4 Orchid Orthopedics, 5404 Baden-Dätwill, SwitzerlandArmando.Salito@orchid-ortho.com (A.S.); 5 Orchid Orthopedic, Memphis, TN 38118, USA; Prathamesh.Kulkarni@orchid-ortho.com

**Keywords:** Porous Ti-6Al-4V implants, quercitrin, multifunctional coating, ALP activity, osteocalcin release, additive manufacturing, orthopedic implants

## Abstract

(1) One strategy to improve the outcome of orthopedic implants is to use porous implants with the addition of a coating with an antibacterial biomolecule. In this study, we aimed to produce and test the biocompatibility, the osteopromotive (both under normal conditions and under a bacterial challenge with lipopolysaccharide (LPS)) and antibacterial activities of a porous Ti-6Al-4V implant coated with the flavonoid quercitrin in vitro. (2) Porous Ti-6Al-4V implants were produced by 3D printing and further functionalized with quercitrin by wet chemistry. Implants were characterized in terms of porosity and mechanical testing, and the coating with quercitrin by fluorescence staining. Implant biocompatibility and bioactivity was tested using MC3T3-E1 preosteoblasts by analyzing cytotoxicity, cell adhesion, osteocalcin production, and alkaline phosphatase (ALP) activity under control and under bacterial challenging conditions using lipopolysaccharide (LPS). Finally, the antibacterial properties of the implants were studied using *Staphylococcus epidermidis* by measuring bacterial viability and adhesion. (3) Porous implants showed pore size of about 500 µm and a porosity of 52%. The coating was homogeneous over all the 3D surface and did not alter the mechanical properties of the Young modulus. Quercitrin-coated implants showed higher biocompatibility, cell adhesion, and osteocalcin production compared with control implants. Moreover, higher ALP activity was observed for the quercitrin group under both normal and bacterial challenging conditions. Finally, *S. epidermidis* live/dead ratio and adhesion after 4 h of incubation was lower on quercitrin implants compared with the control. (4) Quercitrin-functionalized porous Ti-6Al-4V implants present a great potential as an orthopedic porous implant that decreases bacterial adhesion and viability while promoting bone cell growth and differentiation.

## 1. Introduction

There is a need to improve orthopedic implants’ lifetime due to the increase in life expectancy and the increasing popularity of extreme sports, as well as the reduction of production costs of implants [1]. To increase bone-to-implant integration, the use of porous materials with lower Young modulus than nonporous implants is being evaluated, giving space for cell adhesion, bone growth, and in vivo vessel formation while preventing bone atrophy [1,2].

Twenty percent of implant failures are attributed to infection, *Staphylococcus aureus* and *Staphylococcus epidermidis* accounting for around 70% of orthopedic infections [3]. In addition, implant-associated infections are often recurrent, expensive to treat, and associated with high rates of morbidity, if not mortality. The microbial colonization of the implant surface allows the formation of a biofilm, which is difficult to treat with antibiotics, creating the need of additional surgeries, and sometimes the replacement of the implant [4]. With the rising of antibiotic resistances, there is a pressing need for finding new alternatives to antibiotic-coated surfaces, specially coatings that can be adapted to porous surfaces. The use of silver in orthopedic implants has been hampered due to its known toxicity for eukaryotic cells; depending on silver concentration and the release of silver ions, which could affect osseointegration, and the rising of bacterial silver resistance [3,5]. In fact, in vivo studies evaluating different silver coatings have shown either no differences or decreased bone formation [6,7,8]. Other studies have focused on the coating of the surfaces with molecules that improved osteoblast proliferation and differentiation without testing bacterial response [9,10]. The development and application of multifunctional coatings that could inhibit bacterial cell adhesion while still promoting osseointegration could be a promising approach as these two issues are intimately related [3,4]. In previous studies, we searched for agents under investigation for osteoporosis treatment, selecting quercitrin as a good candidate for its properties on bone formation and decreasing *S. epidermidis* growth rate [11,12]. Flavonoids are synthesized by plants in response to microbial infection, having shown antibacterial and anti-inflammatory properties among others, and the ability to reverse antibiotic resistance and enhance the action of the current antibiotics [13].

In previous studies in our research group, a coating method by wet chemistry using the flavonoid quercitrin was developed on nonporous coin implants [14]. We demonstrated that quercitrin-coated surfaces are bioactive, presenting osteogenic, osteopromotive, antifibrotic, and antibacterial properties [15,16]. In this study, we aimed to produce and test the biocompatibility and bioactivity of quercitrin-coated porous Ti-6Al-4V implants on osteoblastic cells and *S. epidermidis* to demonstrate the multifunctional properties of the coating.

## 2. Methods and Materials

### 2.1. Porous Implants Production and Characterization

Ti-6Al-4V implants of 6.25 mm of diameter and 5 mm of total height containing 3 mm in thickness of a porous structure made by 3D Additive Manufacturing with Arcam Electron Beam Technology provided by Orchid Orthopedics (Memphis, TN, USA) (control group) were used for the study.

Structure of Ti-6Al-4V porous implants were characterized using a Hyperion X9 cone beam computed tomography (CBCT) Scanner (MyRay, Cefla s.c. Imola, Italy). A 3D morphometric analysis was conducted with the CTan software (Skyscan, Aartselaar, Belgium) to determine the porosity, pore size, structure thickness, and pore size distribution.

### 2.2. Implant Coating

**Figure 1:**
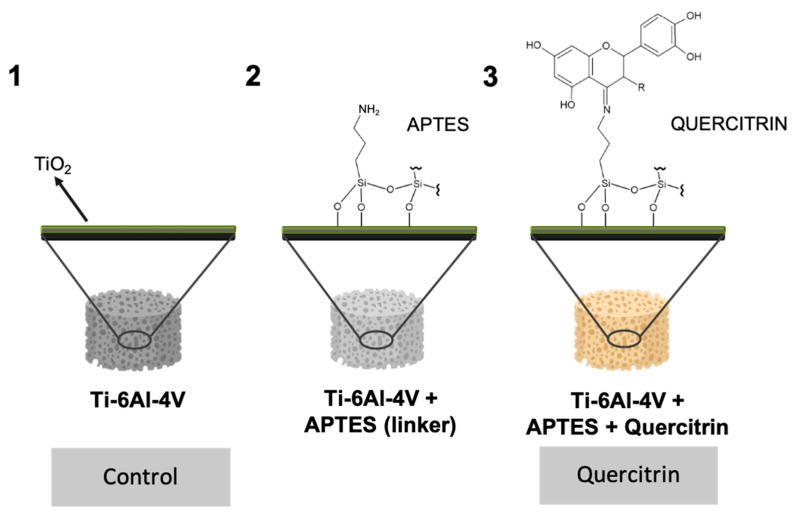
Schematic representation of quercitrin coating on Ti-6Al-4V porous implants, and experimental groups used in the study, control and quercitrin-coated implants.

To obtain the quercitrin-modified surfaces in the full porous structure generated by 3D manufacturing, Ti-6Al-4V implants were modified as previously described with few modifications (Figure 1) [15]. Briefly, to obtain the quercitrin-modified surfaces, Ti-6Al-4V implants were passivated with 30% HNO_3_ for 30 min, rinsed with water until the pH became neutral, and left in water for 48 h. Then, implants were dried under a N_2_ flow and aminosilanized with 2% (3-Aminopropyl)triethoxysilane (APTES) in dry toluene for 24 h under a controlled atmosphere. After that, the surfaces were chemically functionalized with quercitrin by immersion in a 1mM aqueous solution at pH 5.5 for 1 h. The water pH was previously adjusted with HCl until it reached 5.5, and then the 1mM quercitrin solution was prepared. Then, samples were washed twice with water, dried under a N_2_ flow, and stored under vacuum at −20 °C until use. The samples were prepared in aseptic conditions.

### 2.3. Mechanical Characterization of Coated and Uncoated Implants

Two coated and two noncoated samples were used for compressive tests. A Universal Testing Machine (Zwick/Z100, Ulm, Germany) was set at a crosshead speed of 1 mm/min, and the 1000 N load was applied, using a compression plate bigger than the sample. The measurements were made at room temperature. The elastic modulus was determined as the slope of the linear portion of the stress–strain curve.

### 2.4. Visualization of Quercitrin Coating Onto the 3D Implants by Confocal Microscopy

The method uses 2-aminoethyl diphenylborinate (DPBA; Sigma Aldrich, St. Louis, MO, USA), a flavonoid-specific probe that spontaneously forms a fluorescent complex with flavonoid compounds. Briefly, 3D coated and uncoated implants were carefully stained with 20 μL of 22 mM DPBA in methanol, followed by 20 μL 5% (m/v) PEG4000 (Sigma-Aldrich) in ethanol. After 1.5 h, fluorescence images (λex = 450−490 nm) were taken with a confocal microscope (Leica DMI 4000B equipped with Leica TCS SPE laser system, Wetzlar, Germany).

### 2.5. Cell Culture

The mouse osteoblastic cell line MC3T3-E1 (DSMZ, Braunschweig, Germany) was routinely cultured at 37 °C in a humidified atmosphere of 5% CO_2_, and maintained in minimum essential medium alpha modified (α-MEM) supplemented with 10% fetal bovine serum (Biowest, Nuaillé, France), penicillin (100 µg/mL) and streptomycin (100 µg/mL) (Biowest, Nuaillé, France).

For the experiments, 1 × 10^5^ cells/implant were seeded, and in order to guarantee a homogeneous cell distribution inside the implants, an agitated seeding method was used [17]. Throughout the duration of the experiment, cells were maintained static at 37 °C in a humidified atmosphere of 5% CO_2_ and media was replaced 3 times per week.

For the experiment under bacterial challenging conditions, immediately after seeding, 1 µg/mL lipopolysaccharide (LPS) (*Escherichia coli* O111:B4; Merck, Darmstadt, Germany) was added to the cells.

### 2.6. Determination of Cell Viability: LDH Activity

Lactate dehydrogenase (LDH) activity in the culture media 48 h after seeding was used as an index of cell death following the manufacturer’s kit instructions (Roche Diagnostics, Mannheim, Germany). The media of coated and uncoated implants was evaluated, and results were presented relative to the LDH activity in the medium of cells seeded on plastic (low control, 0% of cell death) and on plastic adding 1% triton X-100 (high control, 100% of death), using the following equation: Cytotoxicity (%) = (exp.value – low control)/(high control – low control) * 100.

### 2.7. Immunofluorescence

Cells grown for 48 h on the implants were fixed for 15 min with 4% formaldehyde in phosphate-buffered saline (PBS) at room temperature. For nuclei visualization, cells were stained with Phalloin-Fluorescein Isothiocyanate (FITC) and 4’,6-diamidino-2-fenylindol (DAPI) (Sigma, St. Louis, MO, USA). Then, coverslips were mounted with fluorescent mounting medium (Daco Cytomation, Carpinteria, CA, USA). Two samples of each group were used to perform the experiment, and four images of each sample were taken with the confocal microscope (Leica DMI 4000B equipped with Leica TCS SPE laser system, Wetzlar, Germany). Nuclei were counted using Image J software (National Institutes of Health, Bethesda, MD, USA).

### 2.8. Alkaline Phosphatase (ALP) Activity

Cell were collected after 14 days of culture on coated and noncoated porous implants to determine alkaline phosphatase (ALP) activity. PBS containing 0.1% Triton X-100 was added to disrupt cell membrane and solubilize proteins. Cell lysates were sonicated to improve protein recovery. After centrifugation at 18,000× *g* for 5 min at 4 °C, the supernatants were acquired and assayed for ALP activity as previously described [18]. ALP activity was calculated by measuring the cleavage of p-nitrophenyl phosphate (pNPP) (Sigma, St. Louis, MO, USA) in a soluble yellow end product, which absorbs at 405 nm. In parallel to the samples, a standard curve with calf intestinal alkaline phosphatase (CIAP) (Promega, Madison, WI, USA) was constructed; 1µL from the stock CIAP was mixed with 5 mL of 0.1% Triton X-100 in PBS (1:5000 dilution) and subsequently diluted 1:5.

### 2.9. Osteocalcin Content

Osteocalcin (also known as bone gamma-carboxiglutamic acid—BGLAP) content in the culture media was determined by ELISA following the manufacturer’s instructions (ABEXA abx576005, Houston, TX, USA).

### 2.10. Staphylococcus epidermidis Live/Dead Ratio and Adhesion

*S. epidermidis* 4184 (Colección Española de Cultivos Tipo, Valencia, Spain) were grown from frozen stocks in lysogeny broth. After an overnight incubation, 0.8 mL bacterial suspensions (1.9 × 10^7^ UFC/mL) were incubated for 4 h with the implants. Samples were incubated with temperature control and shaking function at 37 °C. Live/Dead ratio of bacteria was determined using the LIVE/DEAD BacLight bacterial viability kit (Invitrogen, Thermo Fisher Scientific, Waltham, MA, USA), following manufacturer’s instructions.

For bacterial adhesion, samples were fixed in 4% glutaraldehyde in PBS for 2 h and washed with distilled water. Then, samples were dehydrated with the addition of 50%, 70%, 90%, and 100% ethanol solutions in 30 min intervals. The remaining ethanol was evaporated before sputter gold coating. A scanning electron microscope (SEM; Hitachi S-3400 N, Krefeld, Germany) in secondary electrons, low-vacuum conditions, and 15 kV of voltage was used to acquire images. Six samples of each group were used to perform the experiment, and five images of each sample were taken and analyzed using ImageJ software (National Institute of Health, Bethesda, MD, USA).

### 2.11. Statistics

All data are presented as mean values (SD). A sample size of six was calculated using G Power (version 3.1.9.4 downloaded from the Heinrich Heine Universität Düsseldorf page), using data from previous experiments with an effect size (d) of 2 and a power of 0.8. The Shapiro–Wilk test was done to assume parametric or nonparametric distributions for the normality tests; all the data was parametric. Differences between groups were statistically compared by Student’s *t*-test. Results were considered statistically significant at *p*-values <0.05. SPSS^®^ program for Windows, version 17.0 (SPSS Inc, Chicago, IL, USA) was used.

## 3. Results

### 3.1. Porous Ti-6Al-4V Implants Characterization

**Figure 2:**
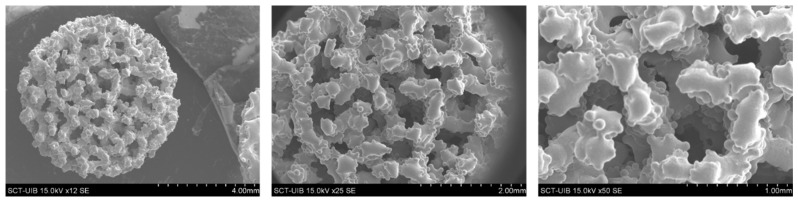
(*n* = 2). Scanning electron microscope images of 3D uncoated implants. Images show the 3D implants used for the study at different magnification. Scale bars are showed in each image.

Implant morphology was visualized by SEM (Figure 2), and the porosity analyzed using CBCT analysis. Implants presented a porosity of 51.7% ± 2.7% with a pore size of 488.9 ± 11.1 µm and a structure thickness of 443.7 ± 15 µm. In addition, pore size was distributed as follows: 150 µm, 5.9%; 300 µm, 16.2%; 450 µm, 32.7%; 600 µm, 36.7%; and 750 µm, 8.6%. The quercitrin coating did not change pore parameters. However, we wanted to study if the coating process had any effect on the mechanical properties of the implants. We found that the Young modulus for control and quercitrin-coated 3D implants was 5.18 ± 0.6 and 5.58 ± 0.3 GPa respectively, with no statistical differences.

**Figure 3:**
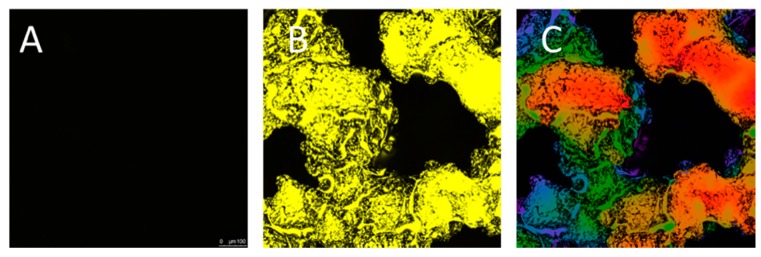
Confocal micrographs of DPBA (2-aminoethyl diphenylborinate) staining of 3D implants (*n* = 2). **A**: Control uncoated 3D implant. **B:** Quercitrin-coated 3D implants. **C:** Image showing a depth projection micrograph of a Quercitrin-coated implant, images are 2D reconstructions of sections acquired repeatedly in sequential steps along the *z*-axis. The color code corresponds to the *z*-axis depth of DPBA fluorescence, coded from blue at 0 µm and pink at 500 µm depth.

Next, quercitrin coating on 3D implants was imaged by confocal microscopy. While no staining was observed on the control uncoated 3D implants (Figure 3A), a homogenous coating was obtained over the whole 3D implant, as shown by the depth projection micrographs of 500 µm, ranging from blue at 0 µm to pink at 500 µm depth (Figure 3B,C).

### 3.2. Biocompatibility and Bioactivity of the Implants

**Figure 4:**
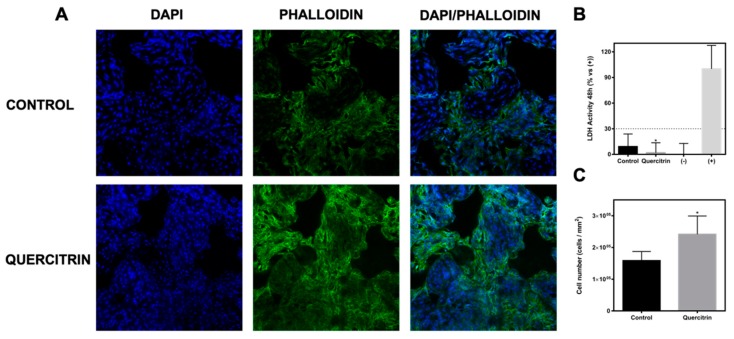
Biocompatibility of the porous implants. **A)** Confocal micrographs of MC3T3-E1 cells cultured for 48 h on 3D implants. Cells were stained with Phalloidin-FITC (stains actin fibers) and DAPI (stains nucleus) (*n* = 2). **B)** Lactate dehydrogenase (LDH) activity measured in culture media 48 h after seeding onto the 3D implants. Low control (-, 0% toxicity) was obtained from cells seeded on plastic. High control (+, 100% toxicity) was obtained from culture media of cells seeded on plastic and treated with 1% Triton X-100 (*n* = 12). **C)** Number of cells growing on 3D implants 48 h after seeding. Cells were stained with DAPI and counted with Image J software (*n* = 2 replicates, 4 images of each sample). Values represent the mean (SD). Results were statistically compared by Student’s *t*-test: * *p* < 0.05 vs. Control.

The biocompatibility was evaluated by the LDH assay that detects the amount of LDH that leaks out through the plasma membrane of damaged cells, as a marker of cytotoxicity. As it can be observed in Figure 4B, after 48 h of cell culture, both control and quercitrin groups presented LDH activity values lower than 30%, which is the maximum value accepted for cytotoxicity of medical devices according to ISO-10993:5. Moreover, lower LDH activity was found for the coated group, indicating a better biocompatibility of the quercitrin-coated 3D implants. Additionally, a higher number of cells on the quercitrin group was found 48 h after seeding (Figure 4C), as observed on the confocal microscopy images (Figure 4A).

**Figure 5:**
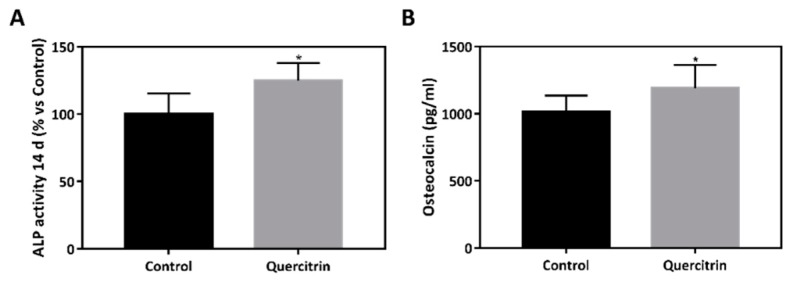
Bioactivity of the porous implants. **A)** Alkaline phosphatase activity of MC3T3-E1 cells seeded onto the 3D implants after 14 days of culture (*n* = 12). **B)** Osteocalcin levels released to culture media of MC3T3-E1 cells cultured onto the 3D implants at 14 days of culture (*n* = 12). Values represent mean (SD). Results were statistically compared by Student’s *t*-test: * *p* < 0.05 vs. Control.

Bioactivity of the implants was further evaluated with two differentiation markers of bone cells, ALP activity and osteocalcin production. ALP is an early marker of osteoblast differentiation that is involved in hydroxyapatite crystal deposition [19]. On the other hand, osteocalcin is produced by osteoblasts and it is implicated in mineralization. Higher ALP activity and osteocalcin release was observed in the quercitrin group compared with control after 14 days of culture (Figure 5A,B), indicating higher osteoblast differentiation.

### 3.3. Bioactivity of the Implants under Bacterial Challenging Conditions

**Figure 6:**
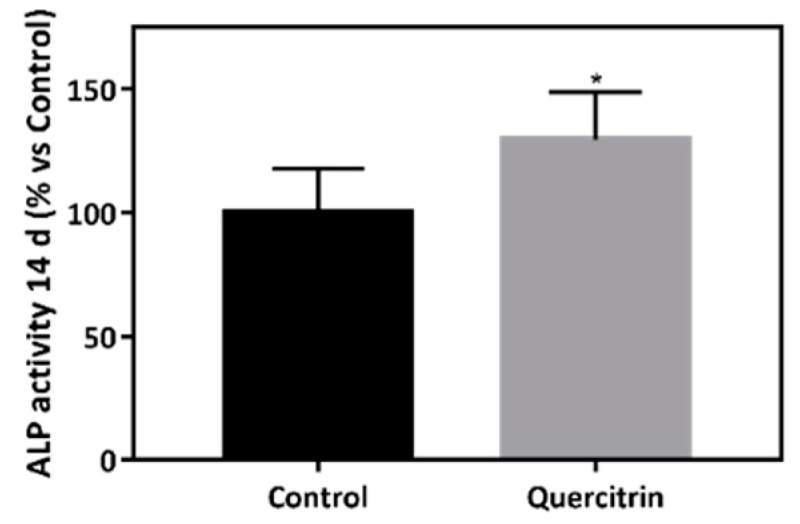
Implant bioactivity under bacterial challenging conditions. Alkaline phosphatase activity in MC3T3-E1 cells treated with 1µg/mL *E. coli* LPS (lipopolysaccharide) for 72 h seeded onto coin and 3D implants at 14 of cell culture (*n* = 6). Values represent mean (SD). Results were statistically compared by Student’s *t*-test: * p < 0.05 vs. Control.

To simulate the conditions induced under a bacterial infection, cells were treated with 1μg/mL of LPS. Lack of cytotoxicity was confirmed by LDH activity (data not shown). Cells cultured onto 3D implants coated with quercitrin showed increased ALP activity after 14 days of culture compared with control (Figure 6).

### 3.4. Effect of the Implants on S. epidermidis Survival and Adhesion

**Figure 7:**
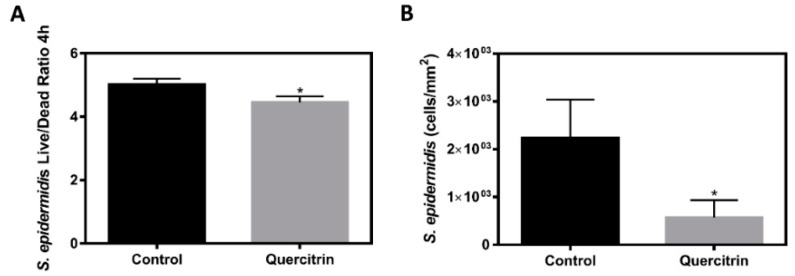
Effect of the implants on *S. epidermidis* survival and adhesion. **(A)** *S. epidermidis* Live/Dead Ratio cultured for 4 h on the implants (*n* = 6). **(B)** *S. epidermidis* adhesion on the implants after 4 h of incubation (*n* = 6). Values represent mean (SD). Differences between groups were assessed by Student *t*-test: * *p* < 0.05 vs. control.

Finally, *S. epidermidis* Live/Dead ratio and adhesion after 4 h was analyzed to test the effect on bacterial survival of the functionalized surface. Figure 7A shows a lower Live/Dead ratio on the Quercitrin-functionalized surface, which indicates that it decreases bacterial survival, promoting bacterial death and reducing the number of bacteria adhered to the surfaces after 4 h (Figure 7B).

## 4. Discussion

There is an urgent need of establishing a pathway for effective modification of porous metal implant surfaces to achieve improved osseointegration and reduced risk of infection that can be readily translated into several orthopedic or dental clinical applications. Among the different strategies to achieve this goal, we selected a chemically bonded coating with a natural biomolecule rather than the use of other biological enhancement strategies (e.g., cell seeding) or physical treatments (e.g., roughness).

The selection of this strategy responds to several issues that are important for bridging the gap between basic and translational research, with new implants that can reach the market in the following next years. One of these criteria is related to the production of this coating (low cost, simple and easy to scale-up, reproducible, good availability, easy handling, retention of physical properties of the metal porous alloy), which effectively produces a homogeneous, thin nanocoating with quercitrin covalently immobilized using its carbonyl group and a silane linker through all the depth of porous Ti-6Al-4V implants, by using a wet chemistry method [14]. Using fluorescence confocal microscopy, we were able to check the presence of quercitrin not only in the surface of the implant but up to 500 µm depth. The thickness of the coating was not analyzed, but according to other studies it should be in the nanoscale level (50–100 nm) [15,20,21]. Another factor is related to the desired features of a bone porous implant, i.e., an open interconnected network, with a pore size >300 µm, pore interconnects >100 µm, and mechanical properties similar to bone. Thus, the quercitrin porous implants did not alter these structural characteristics after the coating and showed a Young Modulus similar to control implants (between 5–6 GPa), which is lower than the one reported for Ti-6Al-4V solid implants (114 GPa) [10,22], but closer to cortical bone (10-30 GPa) [1,2]. Like this, the development of porous materials with decreased rigidity could prevent the shielding effects and bone atrophy that can lead to the implant loosening. For clinical use, the coating can be pasteurized (90 °C, 30 min) [16] or sterilized by X-ray dose of 25 and 45 KGy (unpublished results).

Another important factor is the biocompatibility of the resulting coated porous implants. In agreement to previous studies [14,15], quercitrin-coated implants were more biocompatible and presented a higher proliferation of bone cells (52% more) than control implants. Flavonoids are considered nontoxic because of their ubiquity in all sorts of plant-derived foods and beverages, and it is also related to their antioxidative, anti-inflammatory, antimutagenic, and anticarcinogenic properties [23]. These natural products are well known for their beneficial effects on health, and efforts are being made to isolate the flavonoids from plants, as an indispensable component of many nutraceutical, pharmaceutical, medicinal, and cosmetic applications.

Moreover, the fact that flavonoids have multifunctional properties (i.e., osteopromotive, anti-inflammatory, antimicrobial) makes this biomolecule an excellent choice as coating on orthopedic and dental implants. Other antibacterial strategies such as Ag coating on porous implants have been tested, but in vivo results did not show antibacterial effects but increased inflammation and osteoclast formation [6,7,8], which shows the importance of finding an antibacterial coating that induce a good tissue response. Using our quercitrin coating on a 2D titanium implant in a previous study, we demonstrated that this coating has good biocompatibility and decreases osteoclast function in a rabbit tibia model [24]. This fact, together with the osteopromotive properties (increased ALP activity and osteocalcin secretion) found in this study in the quercitrin-coated porous Ti-6Al-4V implants and in agreement with previous studies [14,15], makes this coating attractive for further testing, waiting to confirm the results in an in vivo study with porous implants in both healthy and infected conditions. We are planning to evaluate the anti-inflammatory, antibacterial, and osseointegration properties of the implants. Thus, besides the evaluation of implant osseointegration, the presence of bacteria and infiltrated inflammatory cells in histological sections as a response of the infection will also be studied.

Another important point is to test the coating bioactivity under bacterial challenging conditions, using an LPS stimuli that increases inflammation [25], to verify if the osteoblast function is maintained. According to the anti-inflammatory properties of the flavonoids, ALP activity of osteoblasts on quercitrin-coated implants was higher than on the control, with an increase comparable to the results with the nonstimulated conditions, indicating that the osteopromotive effect was maintained even under inflammatory conditions. This anti-inflammatory effect of the coating has also been demonstrated in a previous study using human gingival cells with increased collagen levels and lower prostaglandin E2 release [16].

Last, due to the clinical implications of implant-related infections and with the rising of antimicrobial resistance, there is a need to develop antibacterial coatings with other molecules rather than antibiotics, although the effectiveness of antibiotic coatings to decrease bacterial load on the implants has already been demonstrated [26,27]. To test the antiadhesive effect of the quercitrin-coated Ti-6Al-4V implants, we used *Staphylococcus epidermidis*, which is present in the skin and involved in many implant-related orthopedic infections. *S. epidermidis,* like *S. aureus,* does not produce many toxins and exoenzymes that damage the tissue, but the success of *S. epidermidis* is due to its ability to adhere to surfaces and remain there, under a protection of a biofilm. Thus, it is important to highlight that with the quercitrin coating we were able to decrease bacterial adhesion by 75% and also produce a bactericidal effect, in agreement with previous results [12,16]. In order to avoid the limitations of studying bacterial adhesion using SEM, several areas of each implant were analyzed, allowing us to have an idea of the overall implant. Notably, plants synthesize flavonoids in response to microbial infection, and these compounds have been found to be potent antimicrobial agents against a wide range of pathogenic microorganisms, including *S.aureus* and *S.epidermidis*, and they have also been proposed as resistance-modifying agents that can act synergically with antibiotics against resistant bacterial strains. The exact mechanism of action of quercitrin has not yet been elucidated; however, it has been described that quercetin can cause a decrease in the proton-motive force in *S. aureus* and that an increased membrane permeability can contribute to a synergistic effect with antibiotics [13].

To determine if quercitrin-coated porous implants are effective antimicrobials in the in vivo environment remains crucial in order to confirm the value of our findings since this is an exploratory study.

## 5. Conclusions

Quercitrin-functionalized porous structure of Ti-6Al-4V orthopedic implants produced by additive manufacturing have demonstrated to maintain the mechanical properties of the Young modulus of the implants. Furthermore, the quercitrin coating shows good cell biocompatibility, promotes bone cell growth and differentiation, while decreases bacterial adhesion and viability. All in all, quercitrin-functionalized implants represent a multifunctional coating to simultaneously promote osseointegration and prevent infection of orthopaedic implants, which may be considered in the future as an alternative of hydroxyapatite coating with an additional potential reduction of implant infection.
